# Data on performance and variation index for shield tunnelling through soft deposit

**DOI:** 10.1016/j.dib.2021.107103

**Published:** 2021-04-27

**Authors:** Tao Yan, Shui-Long Shen, Annan Zhou, Hai-Min Lyu

**Affiliations:** a Department of Civil and Environmental Engineering, College of Engineering, Shantou University, Shantou, Guangdong 515063, China; b Civil and Infrastructure Engineering, School of Engineering, Royal Melbourne Institute of Technology (RMIT), Victoria 3001, Australia; c MOE Key Laboratory of Intelligence Manufacturing Technology, College of Engineering, Shantou University, Shantou, Guangdong 515063, China; d State Key Laboratory of Internet of Things for Smart City, University of Macau, Macau, China

**Keywords:** Parameters, Energy consumption, Variation index, Construction efficiency

## Abstract

The data presented in this article pertain to field records of EPB shield machine in Metro Line No. 5 in Tianjin, China. Field performance of shield machine (cutterhead, screw machine, and shield advancing) are shown in the figures. Specifically, the database consists of the main parameters for shield tunnelling including cutterhead rotation speed, cutterhead torque, screw machine rotation speed, screw machine torque, shield thrust, and shield advance rate. In addition, the calculation process of energy consumption and variation index R^2^ during the tunnelling are displayed. The value of the dataset is the consideration of silt or clay soil encountered in the shield tunnelling area including the proportion of soils, grain gradation, and effects on performance and energy consumption of different parts in shield machine. These field data are applied to evaluate the construction efficiency in the article titled “Construction efficiency of shield tunnelling through soft deposit in Tianjin” [1].

**Specifications Table**

**Table:** 

Subject area	Civil engineering
More specific subject area	Geotechnical engineering
Type of data	Table, Figure
How data was acquired	Field data and mathematical calculation
Data format	Raw, analysed
Parameters for data collection	The field data were collected in each segment during shield tunnelling by time with the sensors on the shield.
Description of data collection	The energy consumption and variation index were necessary to be calculated in each segment to analyse the construction efficiency of shield.
Data source location	Tianjin City, China
Data accessibility	Data are included in this article and supplementary material in Mendeley Data. doi: http://dx.doi.org/10.17632/r77nc3ft87.1.
Related research article	Yan, T., Shen, S.L., Zhou, A., Lyu, H.M. Construction efficiency during shield tunnelling in soft deposit of Tianjin, China. Tunnelling and Underground Space Technology, doi: https://doi.org/10.1016/j.tust.2021.103917.

**Value of the Data**

• The data of shield parameters can be used to analyse the working performance of shield tunnelling.
• The data of energy consumption during tunnelling process can be used to evaluate shield construction efficiency.
• The calculated variation index of shield tunnelling parameters expresses adaptability of shield tunnelling machine to the soil during tunnelling process.
• The calculation process can help researchers to understand the application of variation index.
• The steps of soil classification algorithm can help researchers to understand the process and the application of K-means algorithm.

## 1 Data Description

**Fig. 1:**
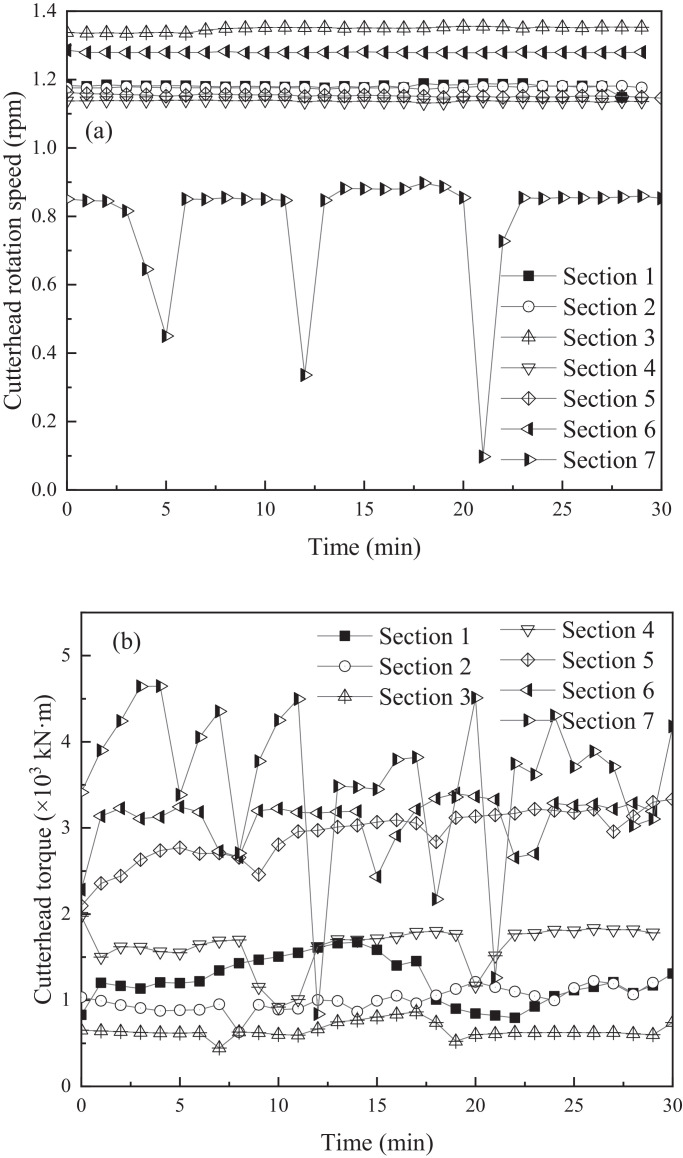
Variations in cutting parameters of cutterhead: (a) real-time cutterheard rotation speed in different sections, (b) real-time cutterheard torque in different sections.

**Fig. 2:**
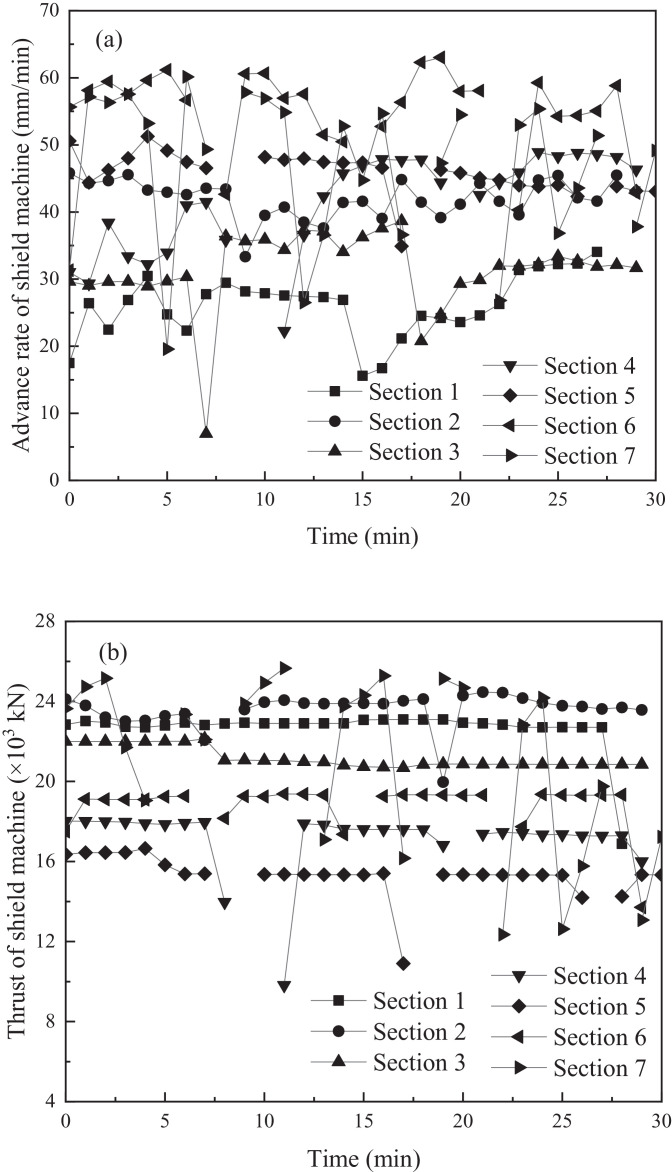
Variations in advancement parameters of shield: (a) real-time advance rate in different sections, (b) real-time thrust in different sections.

**Fig. 3:**
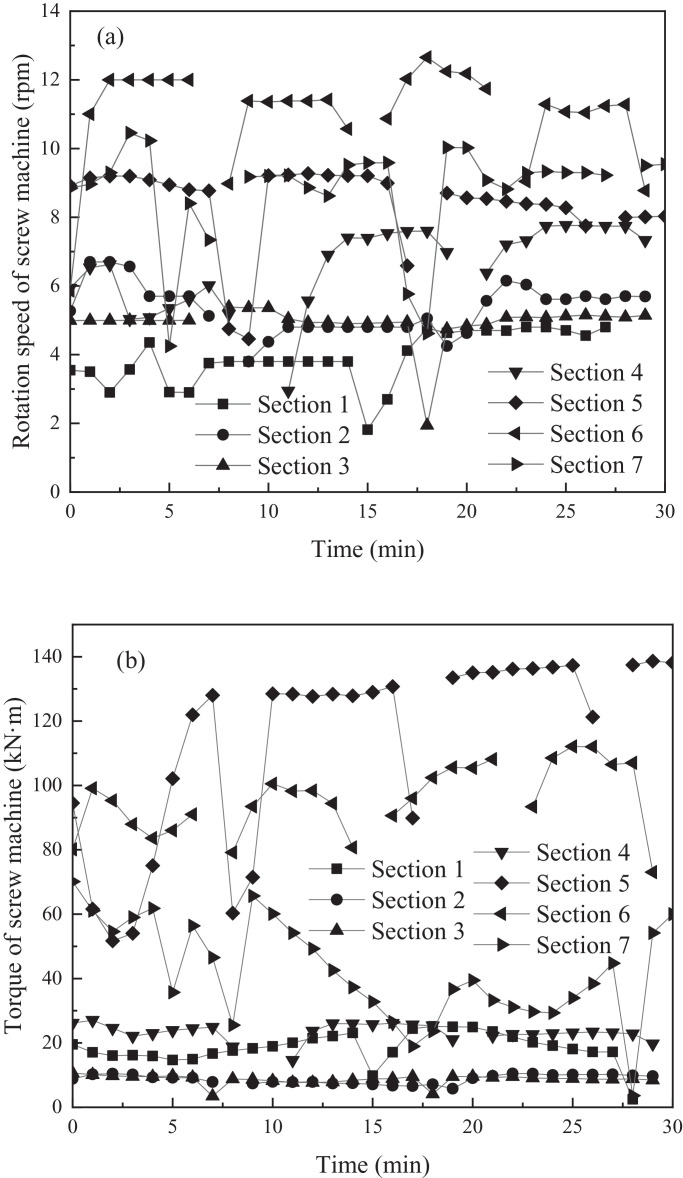
Variations of screw machine parameters: (a) real-time rotation speed in different sections, (b) real-time torque in different sections.

**Fig. 4:**
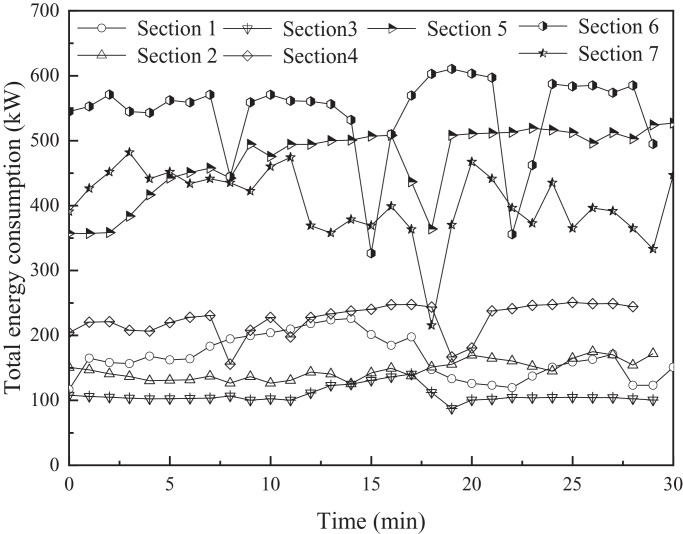
Variations in total energy consumption in different sections.

The data presented here (See Figs. 1–4) was drawn by initial field data from the shield. According to the width of a single segment, seven typical sections with a width of 1.5 m were selected from the boreholes along the design alignment. The soils samples were extracted from the boreholes and tested in the laboratory to obtain their physical and mechanical characteristics. The effective advancement time for each section was approximately 30 min. Each point represented the real-time parameter of the shield machine. These field data were used to evaluate shield construction efficiency and adaptability of shield machine to the soil during tunnelling process. Based on the original data, the energy consumption of shield and variation index during tunnelling are calculated in the supplementary material.

The performance of the cutterhead rotation speed and torque with the advancement time of a segment in different sections are presented in Fig. 1a and b, respectively.

The advance rate and thrust of the shield machine during tunnelling through the respective sections is presented in Fig. 2a and b.

As shown in Fig. 3a and b, the rotation speed and torque of the screw machine were drawn with advancement time at different sections.

The variations in the total energy consumption with time in different sections are presented in Fig. 4. The total energy consumption in Sections 1–4 varied between 100 and 250 kW. The range was lower than that for Sections 5–7 (between 350 and 600 kW). In addition, the fluctuations in the total energy consumption for Sections 1–4 were less than those for Sections 5–7.

## 2 Experimental Design, Materials and Methods

The main energy consumption part of the shield tunnelling includes cutterhead cutting soil, shield advance and screw conveying [1], [2]. The torque and rotation speed can be used to calculate the energy consumption of cutterhead and screw machine [3]. The thrust force and advance rate can be used to calculate the energy consumption of shield advancing. The variation index reflects the degree of deviation between measured and expected values via a calculation of the distance *l_i_* from the measurement point to average point, which is like a variance in statistics. In addition, the parameters of shield machine can be processed and input into the soil classification algorithm to establish a soil classification model. Then, the operation data of the shield machines will be averaged in each ring and used to identify the soil types.

Table 1 gives an example of calculation process of energy consumption and variation index to help researchers to understand the calculation process of energy consumption and variation index. Energy consumption was calculated in terms of cutterhead rotation speed (*n*) and torque (*T*). Variation index was calculated according to maximum value, average value, and the number of cutterhead rotation speed (*n*) and torque (*T*). Table 2 presents the steps of soil classification algorithm including: (1) to select K cluster centres randomly; (2) to calculate the distance between each point and cluster centres; (3) to assign the points to the categories with the smallest distance; (4) to recalculate the cluster centres for each category; (5) to repeat step (3) and step (4) until the cluster centres do not change.

**Table 1:** Example of energy consumption and variation index calculation process.

	*n* (rpm)	*T* (× 10^3^ kN•m)	Energy consumption (kW)	Variation index
1	1.14379	1.12014	$P=\frac{2\times \pi \times 1.14379\times 1.12014}{60}\times 10^{3}\approx 134.10$	$l_{1}=\sqrt{{\left(\frac{1.14379-1.144272}{1.14554}\right)}^{2}+{\left(\frac{1.12014-0.748838}{1.12014}\right)}^{2}}\approx 0.33148$
2	1.14554	0.73108	$P=\frac{2\times \pi \times 1.14554\times 0.73108}{60}\times 10^{3}\approx 87.66$	$l_{4}=\sqrt{{\left(\frac{1.1427-1.144272}{1.14554}\right)}^{2}+{\left(\frac{0.64236-0.748838}{1.12014}\right)}^{2}}\approx 0.09507$
3	1.14392	0.65034	$P=\frac{2\times \pi \times 1.14392\times 0.65034}{60}\times 10^{3}\approx 77.87$	$l_{3}=\sqrt{{\left(\frac{1.14392-1.144272}{1.14554}\right)}^{2}+{\left(\frac{0.65034-0.748838}{1.12014}\right)}^{2}}\approx 0.08793$
4	1.1427	0.64236	$P=\frac{2\times \pi \times 1.1427\times 0.6424}{60}\times 10^{3}\approx 76.83$	$l_{4}=\sqrt{{\left(\frac{1.1427-1.144272}{1.14554}\right)}^{2}+{\left(\frac{0.64236-0.748838}{1.12014}\right)}^{2}}\approx 0.09507$
5	1.14541	0.60027	$P=\frac{2\times \pi \times 1.14541\times 0.6003}{60}\times 10^{3}\approx 71.97$	$l_{5}=\sqrt{{\left(\frac{1.14541-1.144272}{1.14554}\right)}^{2}+{\left(\frac{0.60027-0.748838}{1.12014}\right)}^{2}}\approx 0.13264$
max	1.14554	1.12014	134.1	$R^{2}=\frac{\left(l_{1}^{2}+l_{2}^{2}+l_{3}^{2}+l_{4}^{2}+l_{5}^{2}\right)}{5-1}=0.03612$
average	1.144272	0.748838	89.686

**Table 2:** The steps of the soil classification algorithm.

**Algorithm:** The calculation process of K-means algorithm for soil classification [1]

**Input:** dataset was processed from original data {*x^n^, n* = 1, 2, …, *N*}, where *N* is the number of samples, the dataset contains three columns: lining ring number, TPI, and FPI. [1]
1: Select K cluster centres randomly
2: Calculate the distance between each point and cluster centres:
$D\left(x\right)=\sqrt{{\left(x_{c}-x_{i}\right)}^{2}+{\left(y_{c}-y_{i}\right)}^{2}}$
where (*x_c_, y_c_*) are the coordinates of cluster centre; (*x_i_, y_i_*) are the coordinates of sample point. *x* is the sample point.
3: Assign the points to the categories with the smallest distance
4: Recalculate the cluster centres for each category:
$\mu_{i}=\frac{1}{|C_{i}|}\sum_{x\in C_{i}}x$
where *μ_i_* is the cluster centre of category; *C_i_* is the *i* category; *x* is the sample point in *C_i_*.
5: Repeat step (3) and step (4) until the cluster centres do not change
**Output:** the category of each sample point.

## Ethics Statement

The authors declare that this work does not involve the use of human subjects or experimentation with animals.

## CRediT Author Statement

**Tao Yan:** Conceptualization, Data curtion, Writing – original draft; **Shui-Long Shen:** Funding acquisition; Supervision; Supervision; Writing – review & editing; **Annan Zhou:** Supervision, Data curtion; Writing – review & editing; **Hai-Min Lyu:** Writing – review & editing.

## Declaration of Competing Interest

The authors declare that they have no known competing financial interests or personal relation- ships that could have appeared to influence the work reported in this paper.

## Appendix Supplementary materials

Image, application 1
